# Inferring genetic origins and phenotypic traits of George Bähr, the architect of the Dresden Frauenkirche

**DOI:** 10.1038/s41598-018-20180-z

**Published:** 2018-02-01

**Authors:** Alexander Peltzer, Alissa Mittnik, Chuan-Chao Wang, Tristan Begg, Cosimo Posth, Kay Nieselt, Johannes Krause

**Affiliations:** 1 Integrative Transcriptomics, Center for Bioinformatics, University of Tübingen, Tübingen, 72076 Germany; 2 Department of Archaeogenetics, Max Planck Institute for the Science of Human History, Jena, 07745 Germany; 3 Institute for Archaeological Sciences, University of Tübingen, Tübingen, 72070 Germany; 4 Department of Anthropology and Ethnology, Xiamen University, Xiamen, 361005 China

## Abstract

For historic individuals, the outward appearance and other phenotypic characteristics remain often non-resolved. Unfortunately, images or detailed written sources are only scarcely available in many cases. Attempts to study historic individuals with genetic data so far focused on hypervariable regions of mitochondrial DNA and to some extent on complete mitochondrial genomes. To elucidate the potential of in-solution based genome-wide SNP capture methods - as now widely applied in population genetics - we extracted DNA from the 17th century remains of George Bähr, the architect of the Dresdner Frauenkirche. We were able to identify the remains to be of male origin, showing sufficient DNA damage, deriving from a single person and being thus likely authentic. Furthermore, we were able to show that George Bähr had light skin pigmentation and most likely brown eyes. His genomic DNA furthermore points to a Central European origin. We see this analysis as an example to demonstrate the prospects that new in-solution SNP capture methods can provide for historic cases of forensic interest, using methods well established in ancient DNA (aDNA) research and population genetics.

## Introduction

Advances in modern molecular biology methods and the resulting possibility of extracting genetic information even from ancient specimens, has led to various attempts to reconstruct the genetic legacy of historic individuals. One of the first attempts was made in 2007 on Sven Estridsen, the last Danish Viking king^1^, who died in 1074 AD. Other attempts in reconstructing the genetic legacy of historic individuals include the cases of Francesco Petrarca^2^, the identification of the family of Tsar Nicholas II of Russia^3^, the famous astronomer Nicolas Copernicus^4^, King Richard III of England^5^, the Dark Countess^6^, a proposed blood sample from King Louis XVI king of France^7^ and most recently the Belgian King Albert I^8^. In all of these cases, (except for King Louis XVI, where an Exome and shallow WGS approach was performed), either partial mitochondrial information, such as the hypervariable sequence HVS-I, HVS-II or D-Loop of the mitochondria, or a full mitochondral genome were sequenced. While this is sufficient for investigating maternal ancestry lines, it provides little resolution on genetic origin. Foremost, when focusing on mitochondrial data only, there is no information on the paternal ancestry obtained. Additionally, the prediction of disease risks or phenotypic traits such as hair and eye color are not possible when only mitochondrial information is available. While the availability of cheaper sequencing methods and efficient mitochondrial capture techniques enabled researchers to move from targeting control regions to whole mitochondria, the reconstruction of a full high coverage human genome from ancient human remains via high throughput sequencing still remains costly^9^. In population genetics, where large cohorts of individuals are studied, the cost pressure urged researchers to move on to more cost-efficient and large-scale methods. This has led to the development of specialized in-solution capture methods that target a pre-defined set of SNP positions, as introduced by Haak *et al*.^10,11^. In population genetics of ancient human individuals, these methods are now widely applied to recover population specific diagnostic markers. While these approaches target up to 3.7 M SNP positions^12^ aiming at solely retrieving population diagnostic SNPs in a previously unrivaled resolution, the set of targeted SNPs includes information about various other diagnostic markers as well^13^. This enables a more detailed phenotypic and disease specific analysis of historic individuals on a much broader level than before.

Unlike for population genetics studies, the focus within forensic case studies is shifted to the identification of individuals and prediction of phenotypic traits. In the case of the historical figure focused on in this study, George Bähr, the main goal was to investigate how much information can be retrieved by modern in-solution SNP capture methods for such studies and whether the approach is generally suitable for characterizing historic individuals. George Bähr is most widely known for his work as architect of several churches and in particular the iconic Dresdner Frauenkirche, an important monument in German history due to its destruction in the last few weeks of the Second World War and its recent reconstruction after the German reunification. Born on the ${15}^{th}$ of March 1666 in the village of Fürstenwalde, south of Dresden, as the son of a weaver^14,15^, George Bähr moved to Dresden in 1690 and after several years of work as a carpenter, he was appointed Master Carpenter of the city of Dresden in 1705^16^. During his time there, he was responsible for building both general housing and churches, such as the Orphanage Church in Dresden (1710), the Trinity Church in Schmiedeberg (1713–1716) and several other churches in Forchheim, Königstein, Hohnstein and Kesselsdorf^14^. In 1722, he began work on his most ambitious project, the Dresdner Frauenkirche. In 1730, he was granted the title of Architect for his service to the city of Dresden over the previous decade, including his work on the Frauenkirche^14,15^. Unfortunately, Bähr was unable to see this most prominent piece of work in its full glory, as he died following a pulmonary edema at the age of 72 in 1738, five years before the church was finished^14^. His skeletal remains were initially buried in the Johannis cemetery. However, they were ultimately moved to the crypt of the Frauenkirche in 1854^14–17^, after the cemetery was desecrated and moved to a different location in the city. Unfortunately, there are no written excerpts or paintings that can be used by historians to gain an impression of the physical and personal appearance of George Bähr. Unlike for other famous architects, such as Matthäus Daniel Pöppelmann of the same century^18^, there is almost no material other than basic family background available for George Bähr. Even the most complete biographical and historical works, such as the ones by Möllering^17^, Fischer^15^ and the most recent biography by Gerlach^14^, including intensive archival research, did not reveal any more detailed information on him. After the reconstruction of the Dresdner Frauenkirche, from 1990 to 2005, parts of his skeletal remains were found. In order to obtain biological information such as physical appearance and potential risk alleles for genetically inherited diseases from this historic person of interest, we were provided by the George Bähr foundation with bone samples from his skeletal remains. Through in-solution capture, we were able to obtain high coverage genome wide data from George Bähr and used that information to reconstruct his genetic ancestry and phenotypic traits such as skin and eye color. In addition, we found about a dozen risk alleles for medical conditions, including some that might have contributed to his death.

## Results

**Figure 1:**
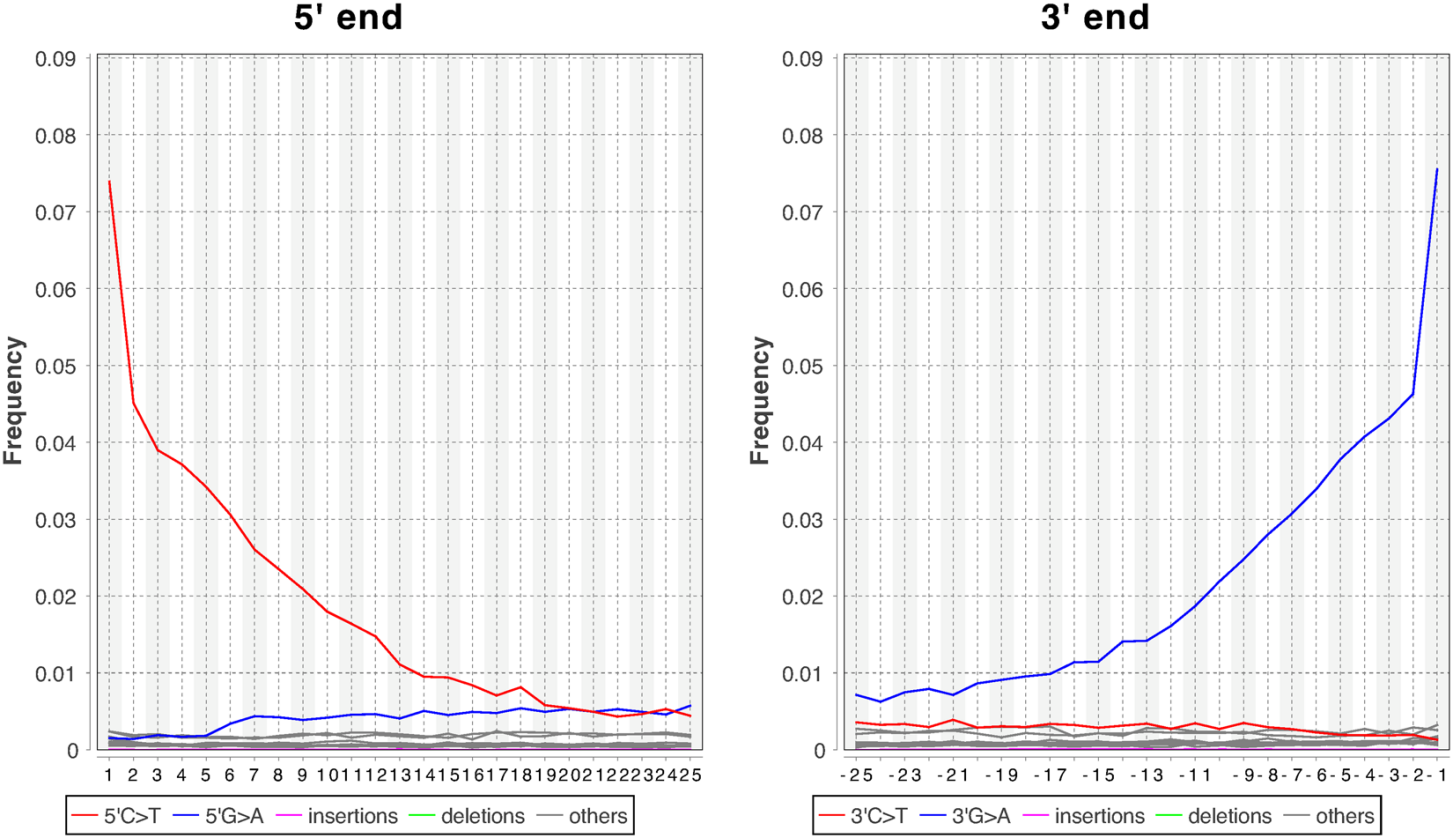
Damage plot for the 5′ and 3′ ends of sequenced reads. Both 5′ and 3′ read ends show $\approx \mathrm{7.5 \% }$ DNA damage on the first respective bases, which is a typical pattern observed for ancient DNA. Since the damage patterns in the initial WGS screening run and the mitochondrial capture experiment are identical, only the WGS screening damage plot is shown here for simplicity. Plots have been created with DamageProfiler.

In total, three independent sequencing experiments were conducted: an initial whole genome shallow shotgun sequencing to determine parameters such as endogenous DNA content, a mitochondrial DNA capture to obtain a full mitochondrial genome and a 390 K SNP capture to obtain high density SNP information on George Bähr. The analysis of the first shallow whole genome shotgun sequencing (WGS), showed a total endogenous DNA content of 62.2%. The mitochondrial DNA capture resulted in a 395 *X* covered mitochondrial genome, accompanied by two high density SNP in solution capture libraries for population and disease specific SNP detection. On the latter, a mean depth of 28.19 *X* coverage on the target dataset of 390 K SNPs published in Haak *et al*.^10^ was achieved, spanning a total of 317,990 SNPs (with ≈80% target efficiency of the capture). The first aim was to authenticate the analyzed DNA to be of historic origin. In order to authenticate the sequenced fragments, the terminal substitution rates were investigated. Typical double stranded aDNA libraries show cytosine to thymine misincorporations at the 5′ end and guanine to adenine misincorporations at the 3′ ends^19,20^. These characteristic substitutions accumulate over time and are caused by deamination of cytosine causing miscoding lesions^21^. As can be seen in Fig. 1, which was created on the intial WGS shallow sequencing run data, up to 7% damage on both 3′ and 5′ ends of the reads can be observed, confirming the presence of ancient DNA.The nuclear 390 K capture libraries were treated with UDG, following a protocol by Briggs *et al*.^20^ to remove damage patterns for improved analysis. The same analysis of the (non-UDG treated) mitochondrial capture library showed identical damage patterns as the initial whole genome shotgun library, as well as minimal mitochondrial contamination as described below, increasing the confidence that the samples indeed contain authentic ancient DNA.

In order to confirm whether the sampled individual was male, a molecular sex determination analysis was done on the sequencing data of the 390 K capture. The results as shown in Table 1 show, that the individual was indeed male.

**Table 1:** Normalized results of sex determination on the skeletal remains of George Bähr. The last column describes the fraction of coverage on the Y chromosome versus the coverage on the autosome. Fu *et al*. reported that a ratio of $\mathrm{ < 0.05}$ can be considered a female individual and a Y-rate $\mathrm{ > 0.2}$ is assured to be a male individual^22^. The results therefore indicate strongly that the investigated individual was male.

Sample	Coverage on chr X	Coverage on chr Y	Autosomal Coverage	${\boldsymbol{c}}{\boldsymbol{o}}{\boldsymbol{v}}({\boldsymbol{Y}})/{\boldsymbol{c}}{\boldsymbol{o}}{\boldsymbol{v}}({\boldsymbol{A}}{\boldsymbol{u}}{\boldsymbol{t}}{\boldsymbol{o}}\mathrm{.)}$
BährAB	10.84	14.68	38.23	0.38

To further exclude a potential contamination of the sampled individual with human DNA from other sources, a mitochondrial contamination test was performed. The estimated mitochondrial contamination was reported to be very low with levels between 0–2%. Quality and authenticity are a major concern in the field of ancient DNA. The last decade has seen a large array of methods to estimate DNA contamination^23^ as well as reliable criteria for authenticity such as DNA damage patterns^21,24^. We followed those criteria strictly and used standard methods to estimate mitochondrial and nuclear contamination rates based on heterozygocity of the mitochondrial genome as well as the sex chromosomes. We can show that the DNA extracted from the remains of George Bähr come from a single male individual that shows damage patterns indicative of at least 100 year old DNA^21^. We therefore conclude the authentic ancient origin of the specimens DNA. A total number of 1,163 known SNPs^25^ on chromosome X covered at least twice were analyzed, resulting in a very low X-chromosomal contamination estimate of 0.003% with an estimated error of $7.391683{E}^{-18}$ ^26^.

After the initial verification and authentication process, the paternal and maternal origin of George Bähr was determined. For this purpose, a complete 395 *X* coverage mitochondrial genome of George Bähr was reconstructed and a quality filtered (q > 30) consensus sequence of his genome was created using schmutzi^27^. His maternal haplogroup was determined to be H35 using Haplogrep 2^28^, which is a common subclade of haplogroup H in Central Europe^29^. Furthermore, the Y chromosomal haplogroup of George Bähr was determined to be R1b1a2a1a2-P312 (Table 2). The assigned Y-chromosomal haplogroup is the most common Y chromosome clade of paternal lineages across much of Western Europe, showing a frequency peak in the upper Danube basin and Paris area with declining frequency towards Italy, Iberia, Southern France and the British Isles^30^.

**Table 2:** Y-Haplotyping results, determined using the ISOGG database.

SNP	Haplogroup	Other Names for SNP	rs ID	Allele Information		
				Y-Position(hg19)	ancestral-derived-Bähr	Depth
P312	R1b1a2a1a2	PF6547; S116	rs34276300	22157311	C-A-A	65
L52	R1b1a2a1a	PF6541	rs13304168	14641193	C-T-T	1
L151	R1b1a2a1a	PF6542	rs2082033	16492547	C-T-T	35
P311	R1b1a2a1a	PF6545; S128	rs9785659	18248698	A-G-G	19
P310	R1b1a2a1a	PF6546; S129	rs9786283	18907236	A-C-C	19
M412	R1b1a2a1	L51; PF6536; S167	rs9786140	8502236	G-A-A	22
L23	R1b1a2a	PF6534; S141	rs9785971	6753511	G-A-A	7
L265	R1b1a2	PF6431	rs9786882	8149348	A-G-G	6
PF6438	R1b1a2	NA	NA	9464078	C-T-T	1
L150.1	R1b1a2	PF6274.1; S351.1	rs9785831	10008791	C-T-T	72
M269	R1b1a2	NA	rs9786153	22739367	T-C-C	1
L320	R1b1a	NA	rs2917400	4357591	C-T-T	1
P297	R1b1a	PF6398	rs9785702	18656508	G-C-C	1

**Figure 2:**
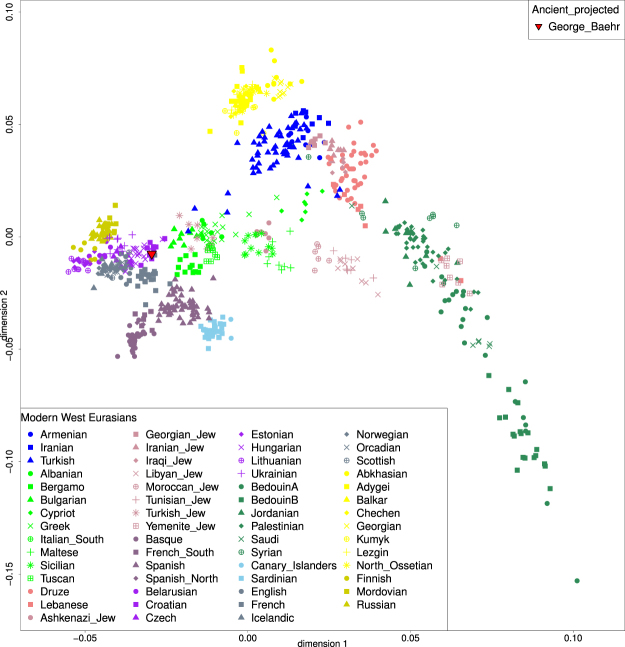
PCA plot generated with EIGENSOFT^31,32^ with representative modern West-Eurasian populations. George Bähr is marked with a red triangle, clustering next to Central and Eastern European populations.

**Figure 3:**
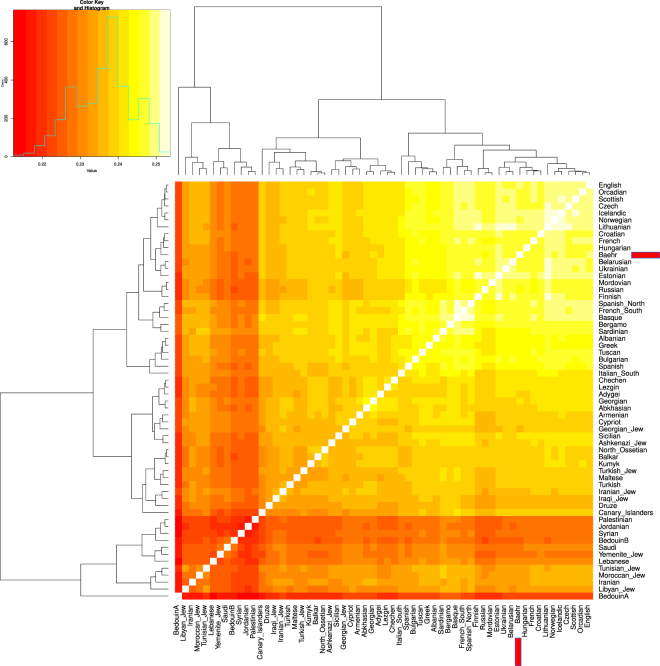
Outgroup ${f}_{3}$ plot for George Bähr. Dark colored areas highlight more distant populations, white highlight closer populations with respect to George Bähr (marked with a red box).

**Figure 4:**
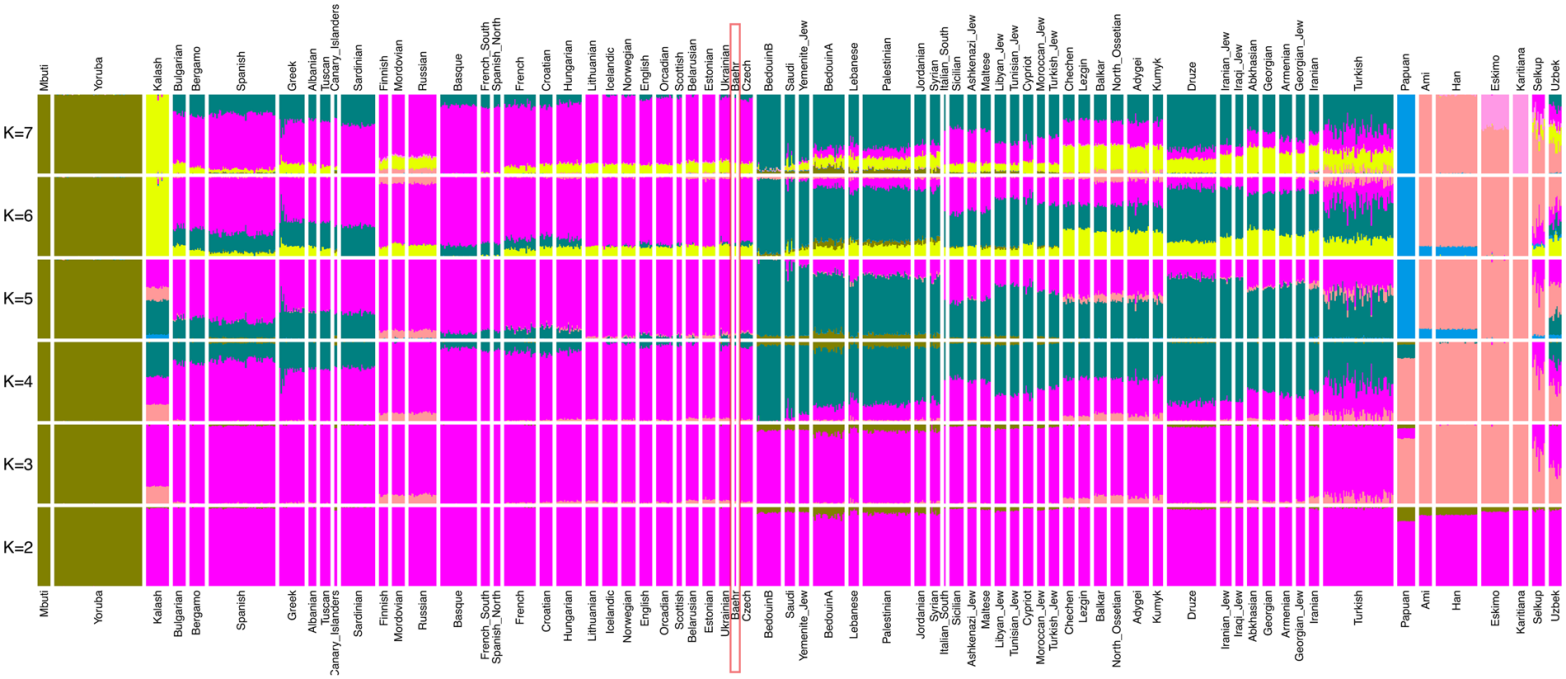
ADMIXTURE graph created with *K* = 7 for the set of elaborated reference populations. George Bähr can be found within the variance of Central European populations, here highlighted with a red rectangle.

A principal components analysis, conducted on 317,990 SNP positions, revealed that George Bähr’s SNP profile matches with profiles commonly found in modern central European individuals as shown in Fig. 2. To further explore the relatedness of George Bähr to European populations, an outgroup ${f}_{3}$ analysis was performed, confirming the initial PCA results, as shown in Fig. 3. To further test whether Africans, South Asians, East Asians, Native Americans and Oceanians share more affinity with George Bähr than with present-day Hungarian, Croatian and French populations, an ${f}_{4}$ analysis was also performed. The statistics as shown in Table 3 imply that there was no extra ancestry from outside Europe in George Bähr. The results from an unsupervised ADMIXTURE^33^ analysis also showed no external genetic components in the genome of George Bähr (Fig. 4).

**Table 3:** ${f}_{4}$statistics results between worldwide populations, Chimp, Europeans and Bähr.

Worldwide populations	Outgroup	Europeans	Bähr	${{\boldsymbol{f}}}_{{\bf{4}}}$	${\boldsymbol{Z}}$
Mbuti	Chimp	Hungarian	Bähr	−0.000108	−0.344
Yoruba	Chimp	Hungarian	Bähr	−0.000187	−0.587
Kalash	Chimp	Hungarian	Bähr	−0.000089	−0.23
Papuan	Chimp	Hungarian	Bähr	0.00052	1.196
Ami	Chimp	Hungarian	Bähr	−0.000055	−0.129
Han	Chimp	Hungarian	Bähr	0.000123	0.297
Karitiana	Chimp	Hungarian	Bähr	0.000451	0.996
Eskimo	Chimp	Hungarian	Bähr	0.000358	0.844
Selkup	Chimp	Hungarian	Bähr	0.000121	0.298
Uzbek	Chimp	Hungarian	Bähr	0.000187	0.488
Mbuti	Chimp	Croatian	Bähr	−0.000041	−0.128
Yoruba	Chimp	Croatian	Bähr	−0.000065	−0.199
Kalash	Chimp	Croatian	Bähr	−0.000078	−0.196
Papuan	Chimp	Croatian	Bähr	0.000558	1.243
Ami	Chimp	Croatian	Bähr	−0.000181	−0.415
Han	Chimp	Croatian	Bähr	0.000032	0.076
Karitiana	Chimp	Croatian	Bähr	0.000315	0.671
Eskimo	Chimp	Croatian	Bähr	0.000281	0.648
Selkup	Chimp	Croatian	Bähr	0.00003	0.072
Uzbek	Chimp	Croatian	Bähr	0.000132	0.335
Mbuti	Chimp	French	Bähr	−0.000008	−0.024
Yoruba	Chimp	French	Bähr	−0.000003	−0.011
Kalash	Chimp	French	Bähr	−0.000064	−0.166
Papuan	Chimp	French	Bähr	0.000524	1.232
Ami	Chimp	French	Bähr	−0.000225	−0.538
Han	Chimp	French	Bähr	0	0.001
Karitiana	Chimp	French	Bähr	0.000202	0.453
Eskimo	Chimp	French	Bähr	0.000153	0.364
Selkup	Chimp	French	Bähr	0.00002	0.051
Uzbek	Chimp	French	Bähr	0.000189	0.498

Next, phenotypically interesting SNPs that are considered to be affected by selection were investigated. With the information obtained by the 390 K SNP capture experiment, George Bähr most likely had brown eyes and light skin, as shown in Table 4. This resembles modern individuals from the same area of Germany, where such a phenotype is commonly found today^34^. Furthermore, Bähr was most likely lactose tolerant as he was heterozygous for the *RS4988235* mutation on the *LCT* gene^35,36^, again a typical phenotype for central Europeans. The 390K SNP capture panel does not include SNPs that can be used to determine hair color.

**Table 4:** Phenotyping results of George Bähr.

SNP	Gene				
	*LCT*	*SLC45A2*	*SLC45A5*	*EDAR*	*HERC2*
	rs4988235	rs16891982	rs1426654	rs3827760	rs12913832
Ancestral	G	C	G	A	A
Derived	A	G	A	G	G
Coverage	$39\times$	$114\times$	8 $\times$	$43\times$	46 $\times$
Derived allele frequency	50%	100%	100%	0%	57%

To ensure consistency, the analysis was limited to high quality bases (*q* > 30) and duplicates were removed after merging of both sequencing libraries. The SNP *RS4988235* in *LCT* is responsible for lactase persistence in Europe^37,38^. Both SNPs at *SLC24A5* and *SLC45A2* are considered to be responsible for light skin pigmentation^39^, whereas the SNP in *HERC2* is the primary determinant of light eye color in present-day Europeans^40,41^. The SNP in the gene *EDAR* affects tooth morphology and hair thickness^42,43^, and was not found to be derived in the investigated sample. All these results were obtained on the 390K SNP capture dataset.

To further elucidate what high density SNP capture methods can provide on such specimen, an extensive literature survey was performed using SNPedia and the database mining tool Promethease^44^. The results of this analysis are shown in detail in Table 5. Several potential candidate mutations were found in George Bähr that are commonly found in modern European populations, such as a variant responsible for the ability to taste bitterness^45,46^. Interestingly, we also found a large number of SNPs associated with modern diseases like Type-2 diabetes, hypertension and coronary artery disease, which could potentially be related^47^ to his reported cause of death, pulmonary edema^14^. Furthermore, a rare variant responsible for age related macular degeneration^48^ was found to be present in George Bähr’s genome.

**Table 5:** Potentially pathogenic and phenotypically relevant SNPs found in George Bähr.

Potentially pathogenic and phenotypically relevant SNPs		
rs ID	Effect	Citation
rs1333049	Associated with coronary artery disease	^49–51^
rs2383206 / rs10757274	Associated with coronary artery disease	^52^
rs5186	7.3x increased risk of Hypertension	^53,54^
rs1061170	5.9x increased risk of age related macular degeneration	^48^
rs1121980	Early onset obesity	^55^
rs1421085	Obesity	^56,57^
rs9939609	Obesity / Diabetes	^58^
rs13266634	Diabetes	^59^
rs4506565	Associated with Diabetes	^60^
rs17817449	Associated with Body weight & increased BMI	^61,62^
rs10246939	Able to taste bitterness	^45,46^

## Discussion

Investigating historic individuals based on genetic data still remains challenging and can only shed light on certain aspects of an individual, such as eye and hair color and a set of well established disease markers. Previous studies on historic individuals^1–6,8^ solely focused on the control region of the mitochondrial DNA and in some cases on full mitochondrial genomes. Although this enabled the analysis of at least the maternal relatedness of historic individuals, the analysis of Y-chromosomal data accompanied by a set of autosomal genetic markers permits researchers to recreate a more detailed genetic picture of historic individuals than before.

Within the scope of this project, a complete mtDNA sequence from the skeletal remains of George Bähr and additionally a set of 317,990 SNPs from his autosomes were retrieved. Standard examination of characteristic damage patterns on the initial shotgun screening data and the mitochondrial capture data suggest an ancient origin for the investigated remains. Very low contamination estimates on mitochondrial and Y-Chromosomal level also showed that the retrieved DNA was authentic and no modern human contamination was found. George Bähr’s maternal haplogroup was determined to be H35 and the Y haplogroup was determined to be R1b1a2a1a2-P312, both commonly found in Central European modern populations. Based on phenotypic analysis, George Bähr had brown eyes, light skin pigmentation and was able to digest lactose in adulthood. The population genetic analysis of ancestry with both ${f}_{3}$ and ${f}_{4}$ statistics as well as an ADMIXTURE analysis on the set of 317,990 SNPs confirmed previous findings on the mitochondrial level: George Bähr was of Central European descent and shared no additional genetic components with populations outside Europe.

Unfortunately, there is not much of a historic record on George Bähr’s private life. Thus any information that can be obtained on a genetic level that elucidates and enlarges information on him could be important, given his contributions to the history of the city of Dresden. Although George Bähr lived a relatively long life given his time period, his cause of death may have been a pulmonary edema as stated by several authors^14^. His genetic make up might have contributed to his death given the detected number of variants found related to obesity, diabetes, hypertension and coronary artery disease, which are now widely seen as high- risk factors for such a cause of death^63^. Although this seems promising in terms of genetic evidence, a direct correlation of such risk factors with an actual cause of death still remains difficult. We see our results as an example of how genome wide information can help to reveal more information on historic individuals for whom scarce or incomplete personal details are available. Written evidence describes that George Bähr’s remains were initially buried at the Johannis cemetery of Dresden and later moved to the crypt of the Frauenkirche in 1854^14–17^. Unfortunately, given the time period of the reburial and the demolished condition of the Frauenkirche after the Second World War, we cannot exclude entirely the possibility of skeletal mixup. However, our reconstructed genetic profile as well as the historical provenience of the human remains suggest that the analyzed specimens indeed belong to George Bähr.

With the rise of cost-efficient in-solution based SNP capture methods, historic samples can now be investigated in a much more detailed way than ever before. In contrast to previous methods that focused on mitochondria or control regions, the additional information obtained using established SNP capture protocols can provide much more information for researchers or historians to investigate more complex forensic, population genetic and medical questions. Although genetic methods with respect to phenotype predictions made some progress in the last few years, one must keep in mind that direct connections between genotype and phenotype are still challenging. Estimating personal characteristics from genetic data, such as the height or appearance of an individual are in their early stages, as shown for example by Mathieson *et al*.^13^. For even more detailed predictions, e.g. facial reconstructions these direct relationships between genotype and phenotype still remain unresolved. Furthermore, the quality of historic genome data is usually inferior to modern genome data and typically introduces additional error sources, rendering statistically profound statements in the context of phenotypic analysis even more complicated.

New and updated capture protocols are incorporating more diagnostic positions and thus provide now even more SNPs for downstream medical and population genetics analysis in the future. We therefore believe that the current SNP capture methods are just the beginning for studies of historic individuals. For example, Mathieson *et al*.^13^ stated that larger cohort studies, such as the one conducted by Mallick *et al*.^64^, could reveal more and more diagnostically relevant SNPs and associations between SNPs that can hopefully help resolve such questions in more detail in future.

## Methods

### Ancient DNA extraction & Initial Screening

Bone samples were taken under standard precaution and clean conditions from the skeletal remains of George Bähr, which had been placed in the crypt of the Dresdner Frauenkirche. We performed DNA extraction and library preparation steps in clean-room facilities. Bone powder was collected using a dental drill and subsequently DNA was extracted using an established protocol^65^. We produced indexed libraries using 20 *μ* aliquot of the generated extract, following the protocol of Meyer *et al*.^66^. Additionally, the libraries were enriched for human mitochondrial DNA in a bead based capture protocol using long-range PCR products as bait for hybridization as introduced by Maricic *et al*.^67^. We included one negative control for every step of DNA extraction and library preparation to ensure consistency of results. DNA sequencing was performed in an initial screening run for the enriched library pools on the Illumina Genome Analyzer IIx platform with $2\times 76+7$ cycles, following the instruction manual for multiplex sequencing (FC-104-400x v4 sequencing chemistry and PE-203-4001 v4 cluster generation kit). In contrast to the manual, the raw reads were aligned to the PhiX 174 reference sequence to obtain training data for a modified base calling application called Ibis^68^. The reads were then filtered according to their individual indices and went into RAW data processing.

### Nuclear 390 K capture

In the clean room facilities of the Institute for Archeological Sciences in Tübingen, Germany, two further libraries from $\mathrm{20\ }\mu l$ extract each were produced in a similar fashion to the screening library, but additionally implementing a UDG and endonuclease $VIII$ damage repair treatment^20^, to remove deaminated bases. The libraries were amplified to reach an amount of about $\mathrm{1,000\ }ng$ DNA for each which was subsequently used in an in-solution hybridization capture approach^11^, targeting a set of $\mathrm{394,577}$ SNPs^10^. DNA sequencing was performed on a HiSeq 2500 with $2\times 101$ cycles.

### RAW data processing and authentication

General RAW data processing for the initial shallow whole genome sequencing (WGS), mitochondrial capture dataset and the 390 K SNP capture data was done using the EAGER pipeline^69^. In all cases, sequence adapters were clipped with Clip&Merge with default settings and the paired end reads were merged respectively. For the initial WGS and the 390 K SNP capture data, the read mapping procedure was performed with BWA^70^ 0.7.15 and reads were mapped against the hg19 human reference genome. For the mitochondrial capture data, reads were mapped against the rCRS reference genome. The CircularMapper approach as implemented in EAGER was used with default settings to increase mapping qualities towards the ends of the utilized mitochondrial reference genome. In all three datasets, WGS, 390 K and mitochondrial capture, DNA damage authentication was performed using our in-house tool DamageProfiler to determine whether characteristic misincorporation patterns of aDNA are present in the investigated datasets^21^. In addition, the mitochondrial data was tested for potential contamination in the EAGER pipeline using schmutzi^27^. On the 390 K capture data, the “MoM” estimate from “Method 1” as well as the “new_llh” X-chromosomal authentication method in ANGSD^26^ was used to quantify potential autosomal contamination on the X chromosome. Furthermore, a molecular sex identification of the remains of George Bähr was performed using the method previously described in Fu *et al*.^22^. This approach calculates the number of reads mapping against the target SNPs on the Y chromosome and compares this to the total number of reads mapping against the target SNPs on the autosome. An empirical threshold from the literature (see^71^) was then used to determine whether the investigated individual was male or female.

### Y-chromosomal analysis

The Y chromosomal haplogroup was determined by examining a set of diagnostic positions on chromosome Y using the ISOGG database version 11.228 (August 19, 2016), utilizing all available positions on the 390 K capture dataset. In order to perform this analysis, the analysis was restricted to reads with a mapping quality higher than 30. Further detailed investigations revealed that mutations separating George Bähr from upstream Y haplogroups such as R1b1a2a1a (see Table 2) are present. For potential haplogroups within the clade investigated R1b1a2a1, R1b1a2a and R1b1a2 (see Table 2) characteristic mutations were found, which made the placement of George Bähr in Y haplogroup R1b1a2a1a2-P312 most likely.

### Population Specific analysis

#### Principal components analysis

A principal components analysis using the smartpca method available in EIGENSOFT^31,32^ was performed using default parameters and the options lsqproject: YES and numoutlieriter: 0. The investigated sample was projected onto the variation of 777 present-day West Eurasians with $\mathrm{317,990}$ SNPs^10^.

#### Admixture

An ADMIXTURE^33^ analysis was performed after pruning the data for linkage disequilibrium in PLINK^72^ with the parameters–indep-pairwise 200 25 0.4 retaining 181,529 SNPs of the 390 K capture dataset^10^. ADMIXTURE was executed with default 5-fold cross validation, varying the number of ancestral populations between *K* = 2 and *K* = 15 in bootstraps of 100 with different random seeds. Again, 777 modern West Eurasians and individuals from worldwide representative populations such as Mbuti, Yoruba, Han, Papuan, Karitiana, Eskimo, Uzbek, Amim, Selkup and Kalash were used for the analysis. The lowest cross-validation errors were observed with *K* = 7.

#### Outgroup ${f}_{3}$/${f}_{4}$ statistics

Additionally, ${f}_{3}$ statistics of the form ${f}_{3}({\rm{Mbuti}};B\ddot{{\rm{a}}}\mathrm{hr},X)$ were calculated to test which West Eurasian populations share the most genetic drift with George Bähr. This analysis was performed using ADMIXTOOLS^73^ with the parameter settings inbreed: YES, computing standard errors with a block jackknife. For the computation of ${f}_{4}$ statistics of the form ${f}_{4}(\mathrm{Worldwidepopulations},\mathrm{Chimp};\mathrm{Europeans},B\ddot{{\rm{a}}}\mathrm{hr})$ ADMIXTOOLS^73^ was applied and again standard errors were computed with a block jackknife.

#### Phenotypic analysis

After uploading a VCF file^74^ to the respective web service Promethease, a more detailed report is created stating potential causes for diseases as well as phenotypic traits. To ensure that found variants are indeed trustworthy, the IGV tool was used to manually confirm the findings of the method before reporting^75^.
